# Seasonal Variation in Antimicrobial Activity of Crude Extracts of *Psammaplysilla* sp. 1 from Phillips Reef, South Africa

**DOI:** 10.1155/2021/7568493

**Published:** 2021-05-11

**Authors:** Wasswa Cuthbert Kibungu, Justine Fri, Anna-Maria Clarke, Anthony Otigbu, Henry Akum Njom

**Affiliations:** ^1^Microbial Pathogenicity and Molecular Epidemiology Research Group (MPMERG), Department of Biochemistry and Microbiology, Department of Biochemistry &Microbiology, University of Fort Hare, Private Bag X1314, Alice 5700, South Africa; ^2^Department of Microbiology, Faculty of Natural and Agriculture Science, North West University, Private Bag X2046, Mmabatho 2375, South Africa; ^3^Agricultural Research Council, Private Bag X1251, Potchefstroom 2531, South Africa

## Abstract

Marine invertebrates constitute a diverse group of marine organisms beneficial to humanity due to their therapeutic significance. The marine sponge species *Psammaplysilla* sp. 1 was collected from Philips Reef, South Africa, over a four-season period and assayed for antimicrobial potential. The physicochemical parameters of the collection site were also recorded. The sponge crude extracts' antimicrobial activity was evaluated using an agar well diffusion assay against 5 pathogens. Phytochemical screening was conducted to identify the presence of 7 critical phytochemical groups. During the four seasons, the mean water temperature was 17.35°C ± 2.06, with autumn recording the highest (20°C) temperature. Antifungal activity was observed by *Psammaplysilla* sp. 1 (30 mm) against *C. albicans,* and this was higher than that showed by standard drugs ICZ-10 *µ*g (15 ± 0.1 mm), FLU-15 *µ*g (21 ± 0.2 mm), and VCZ-5 *µ*g (17 ± 0.1 mm), respectively. Similar bioactivities were observed seasonally with *Psammaplysilla* sp. 1 (22 mm and 24 mm) during autumn and spring, respectively, against *C. difficile* while only crude extracts collected in spring showed bioactivity against *C. albicans*. *Psammaplysilla* sp. crude extracts showed broad-spectrum bioactivity against all test pathogens. DCM : ME crude extracts tested positive for the presence of 2/7 of the phytochemicals (terpenoids and flavonoids). GC-MS revealed several previously reported biologically active compounds such bicyclo[4.2.0]octa-1,3,5-trien-7-ol and phenol, 2,6-dibromo, some of which have been found in plants. This study revealed that sponge bioactivity is dependent on the season and further validated the antimicrobial potential of South African marine sponges.

## 1. Introduction

Nature has been identified as a good source of vast medically significant compounds for many years [1]. In the last decades, problems with antibiotic-resistant microbes have emerged, with an increased evolution of complex diseases, especially amongst immunosuppressed individuals [2]. Therefore, there is a need to discover and develop novel and highly potent antimicrobial agents as conjugates or alternatives to existing antimicrobial therapy that cannot be overemphasized. The marine environment provides a possible source of antimicrobial compounds due to its vast diversity, with its organisms representing approximately 80% of the world's biota [3]. Marine sponges belonging to the phylum Porifera [4] are significant animals that have been reported to be a source of unique natural products ranging from food, fragrances, pigments, enzymes, inhibitors, insecticides, and essential medicines such as antibacterial, anticancer, antiviral, and antifungal agents [5]. Approximately 10,000 pharmacologically bioactive compounds were successfully reported from marine invertebrates such as tunicates, sponges, soft corals, sea hares, nudibranchs, bryozoans, and sea slugs [6].

Bioactive metabolites obtained from the sponge genera *Helicona*, *Petrosia,* and *Discodema* were reported to be potent anticancer and anti-inflammatory agents, respectively. To further support the antimicrobial potential observed from sponges in previous studies, Lakshimi et al. [7] reported that the sponge *Haliclona exigua* produced promising antifungal compounds against *Candida albicans*, *Cryptococcus neoformans*, and *Aspergillus fumigatus*.

Highly diverse groups of active compounds, such as alkaloids, sterols, and peptides, have been produced from various sponge species [7]. These compounds have been found to possess antibacterial activity against drug-resistant strains of *Staphylococcus aureus*, *Pseudomonas aeruginosa*, *E. coli*, *Neisseria gonorrhoeae*, *Streptococcus pneumoniae*, *Mycobacterium tuberculosis,* and *Helicobacter pylori* [8].

These compounds are mainly produced as defensive mechanisms against microbial pathogens [8], hence the growing pharmaceutical interest of these marine species since the early 1950s as sources of novel bioactive metabolites following the discovery of novel bioactive compounds from algae and sponges [9]. Marine invertebrates have been mainly researched for neurophysiological, anticancer, and antiviral potentials instead of their antimicrobial potentials [9]. There is, therefore, a paucity of data on the antibacterial and antifungal activities of marine invertebrates' compounds. Also, although some marine invertebrates' bioactivity has been evaluated, the effect of seasonal differences on the production or bioactivity of their metabolites has not been reported. Therefore, this study aimed to determine the bioactive potentials of sponge extracts collected from Phillips Reef during the four seasons of the year.

## 2. Materials and Methods

### 2.1. Sample Collection and Taxonomical Identification

**Figure 1:**
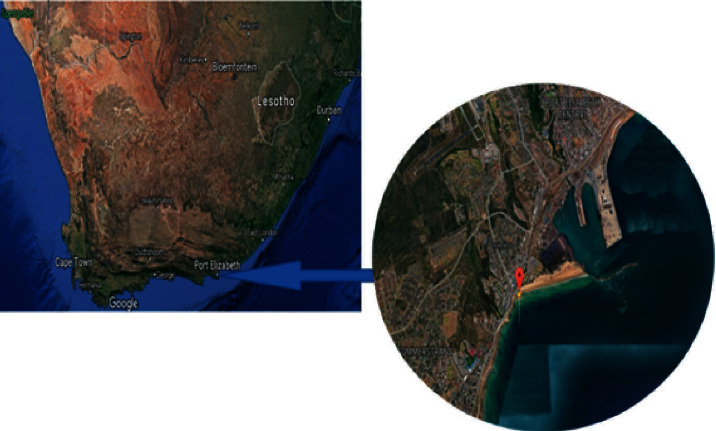
The geographical location of Phillips Reef in Algoa Bay, Port Elizabeth, South Africa.

Samples were collected from Phillips Reef in Algoa Bay with coordinates 33°58'37.2ʺS 25°40'22.8ʺE, Port Elizabeth, Eastern Cape Province of South Africa (Figure 1).

Scuba divers collected *Psammaplysilla sp*. 1 species at depths of 12 m once-off during all four seasons of the year. The site's physicochemical parameters during each collection were recorded using a Conductivity Temperature Depth (CTD) device (Seabird 19plus V2). Each sample was transferred into a sterile zip lock bag containing seawater and was maintained at 4°C. Samples were transported to the Microbial Pathogenicity and Molecular Epidemiology Research Group (MPMERG) Laboratory at the University of Fort Hare for further analysis. Samples that were not analyzed immediately were frozen at −20°C before the extraction of bioactive compounds.

A portion of each sample was sent to the Department of Environmental Affairs Oceans and Coastal Research Centre, Cape Town, and South African Environmental Observation Network, Port Elizabeth, South Africa, for taxonomical identification. Identification was done using morphological approaches. Briefly, a section of each sponge specimen was cut and placed in household bleach to digest the sponge material. The cut sections and spicules preparations were mounted on microscope slides and allowed to dry. Permanent mounts were then made using Entellan or Canada balsam. Sponge morphology, arrangement, and spicule morphology were used for identification.

### 2.2. Bioactive Compound Extraction

Ethyl acetate (EA) and dichloromethane : methanol, DCM : ME (1 : 1), were used for the isolation of bioactive metabolites as previously described [10–13]. For each sponge, 10 g of fresh or thawed sample was minced using a blender and added to 150 ml of each solvent (EA and DCM : ME). The tissue and solvent mixtures of EA and DCM : ME were agitated for 72 and 48 hours, respectively, followed by filtration through a Whatman No.2 filter paper. The filtrates were concentrated using a rotary vacuum evaporator (Lasec Steroglass, Strike 202) at 40°C at 300 rpm. The resulting crude extract residues, approximately 1 ml each per 50 ml of evaporated solution, were stored at 4°C for further analysis.

### 2.3. Test Organisms/Growth

Commercial strains of bacteria, methicillin-resistant *Staphylococcus aureus*, ATCC 49476, and *Pseudomonas aeruginosa* ATCC 10145 were resuscitated on Brain Heart Infusion (BHI) agar (Oxoid, UK) at 37°C for 24 hours. *Clostridium difficile* ATCC 9689 was resuscitated on BHI supplemented with 5 mg ml^−1^ of yeast extract and incubated at 37°C for 48 hours microaerophlically [10]. Fungal species of *Candida albicans* ATCC 10231 and *Aspergillus fumigatus* (ATCC 204305) were cultured on potato dextrose agar (PDA) and incubated at 30°C for three to five days [14].

### 2.4. Antimicrobial Assays

The crude extracts' antibacterial activity was evaluated using the standard agar well diffusion assay on Muller Hinton Agar (MHA) with slight modifications (Selvin and Lipton, 2004). Inoculums of the test bacteria, methicillin-resistant *S. aureus* ATCC 49476, *C. difficile* ATCC 9689, and *P aeruginosa* ATCC 10145 were prepared by transferring colonies from an overnight culture into 0.9% normal saline and turbidity was adjusted to 0.5 McFarland standards (the equivalent of one to two × 10^8^ CFU ml^−1^). Two hundred and fifty microliters of each adjusted bacteria suspension was introduced into 500 ml of MHA (Oxoid, UK), which was cooled to about 40°C poststerilizations.

The mixture was poured into Petri dishes and allowed to solidify. Wells of 6 mm diameter were punched into the MHA plates containing test bacteria using a sterile cork borer. Crude extract concentrations of 80% (v : v) were prepared. This was done in duplicate for all crude extracts obtained using ethyl acetate and dichloromethane : methanol (1 : 1) (v : v).

Twenty microliters of each of the extracts was dispensed into different wells, and a negative control well was filled with the corresponding solvents. Antibiotic disks were also included as positive controls in the assays: vancomycin, VN (30 *µ*g), clindamycin, CD (10 *µ*g), trimethoprim, TM (5 *µ*g), rifampicin, RP (5 *µ*g), and amoxicillin, AMX (25 *µ*g), were tested against MRSA, imipenem, IMP (10 *µ*g), ciprofloxacin, CIP (5 *µ*g), and meropenem, MEM (10 *µ*g), were tested against *P. aeruginosa*, and metronidazole MNZ (5 *µ*g), tetracycline, TE (30 *µ*g), and clarithromycin, CLR (15 *µ*g), were tested against *C. difficile*. *S. aureus* and *P. aeruginosa* inoculated plates were incubated at 37°C for 24 hours while those of *C. difficile* were incubated at 37°C for 48 hours microaerophilically. The resulting diameters of the zones of inhibition were measured in millimeters.

The antifungal property of different extracts against the test fungi, *C. albicans* (ATCC 10231) and *A. fumigatus* (ATCC 204305), was determined as previously reported by Selvin and Lipton [15]. However, the media used were PDA. Negative controls were wells filled with corresponding solvents while the antifungal agents, fluconazole, FLU (15 *µ*g), itraconazole ICZ (10 *µ*g), and voriconazole, VCZ (5 *µ*g), were included as positive controls. Incubation was done at 37°C for three to five days [16]. All assays were done in triplicate, with a total dry weight of 24.5 mg being added to 1 ml of the parent extraction solvent to create a 24 mg/ml stock solution. The quantity selected was selected as it was the average mass of the crude extract powder obtained following rotary evaporation.

### 2.5. Phytochemical Screening of Bioactive Crude Extracts

Crude extracts that exhibited antimicrobial activity against the test bacteria and fungi were tested for phytochemical compounds such as flavonoids, tannins, terpenoids, phenolic compounds, saponins, and alkaloids.

#### 2.5.1. Detection of Flavonoids

Flavonoids were detected using the Juglone test method as described by Sofowara [17]. Diluted ammonia solution was added to crude filtrate followed by adding 1 ml of concentrated sulphuric acid and 2 ml of potassium hydroxide solution and allowed to mix. 1 ml of aqueous filtrate of the sample was added before being observed for visible color changes.

#### 2.5.2. Detection of Tannins

Detection of tannins was carried out as reported by Iyengar [18]. A few drops of 0.1% ferric chloride were added to 0.5 ml of crude filtrate and observed for brownish green or a blue-black coloration which indicated the presence of tannins.

#### 2.5.3. Detection of Terpenoids

The Salkowski test was used to detect terpenoids, as reported by Kiran et al. [19]. To a volume of 5 ml of crude filtrate, 2 ml of chloroform was added, followed by 3 ml of concentrated sulphuric acid. A reddish-brown coloration of the interface indicated the presence of terpenoids.

#### 2.5.4. Detection of Phenolic Compounds

The method reported by Mace [20] was followed. A volume of 2 ml of the crude filtrate and alcohol was mixed with a few drops of neutral ferric chloride (pH 7) solution. A dark green color indicated the presence of a phenolic group.

#### 2.5.5. Detection of Saponins

Saponins were detected using the method described by Venkatesh et al. [21]. In this test, 0.5 ml of latex was dissolved in 5 ml of distilled water in a test tube. The solution was shaken vigorously and observed for a stable, persistent froth with a honeycomb structure.

#### 2.5.6. Detection of Anthraquinones

The crude filtrate (2.5 ml) was shaken with 5 ml of benzene and 2.5 mL of 10% ammonia solution. Pink, red, or violet color indicated anthraquinones as reported by Mujeeb et al. [22].

#### 2.5.7. Detection of Alkaloids

Samples containing alkaloids were identified using the method described by Evans [23]. A volume of 2 ml of 1 M hydrochloric acid was added to 1 ml of the crude filtrate. A few drops of Mayer's reagent (mercuric chloride 1.36 g, potassium iodide 5.0 g dissolved separately, and the volume made up to 100 ml with distilled water) were added to the test tube. A white or creamy precipitate indicated a positive result for alkaloids.

### 2.6. Gas Chromatography-Mass Spectrum Analysis

GC-MS (Shimadzu UV-1800) with NIST library was used to identify the compounds present in the extract. A volume of 3 *µ*l microliters of the EA extract was injected into the GC-MS using a microsyringe. The signals obtained because of the compound elution from the gas chromatography into the detector were represented as peaks. The intensity of the signals and retention time were measured. The retention indices and mass spectra patterns obtained from the detected compounds were compared with those already documented in the NIST library for identity assignment [24].

### 2.7. Statistical Analysis

Means of zones of inhibition obtained from the bioactivity assays were derived and the standard deviations calculated using Microsoft Excel. A two-way analysis of variance (ANOVA) was carried out to determine if there were any statistical differences between the mean zones of inhibition of the ethyl acetate and dichloromethane : methanol crude extracts of *Psammaplysilla* sp. collected during the four seasons (winter, summer, autumn, and spring) against the pathogens of interest. The GraphPad Prism version 7.04 (Prime 7 for Windows) Microsoft Software was used for this analysis. The significance value was set at *p* < 0.05.

## 3. Results and Discussion

### 3.1. Results

#### 3.1.1. Physicochemical Properties of Water Samples at the Site of Sample Collection

The mean water temperature in Phillips Reef during the four seasons was 17.35°C ± 2.06, with autumn recording the highest (20°C) temperature and the winter recording the lowest (16°C). No clear-cut differences in pH recordings were observed for all sampling seasons as the mean pH reading for all four seasons ranged from 8 to 8.78. The same was observed for the salinity readings that ranged between 35.12 psu and 35.47 psu for all four seasons. The average dissolved oxygen (DO) concentration was different across all four seasons, with the winter season recording the highest DO (7 mg L^−1^), while the spring season recorded the lowest DO (5 mg L^−1^). The turbidity of Phillips Reef was high during the spring season, with average turbidity of approximately 3 NTU, while the autumn season recorded the lowest turbidity of approximately 1 NTU. Conductivity was between 43.5 mS cm^−1^ and 48.2 mS cm^−1^ on average across all four seasons. A low coefficient of variability was observed for all parameters during the four seasons (Table 1).

#### 3.1.2. Taxonomic Identification of Marine Species

The collected specimen was identified as *Psammaplysilla* sp. 1 based on morphological appearance. Analyzed specimens were deposited at the Department of Environmental Affairs Oceans and Research facility in Cape Town, South Africa.

#### 3.1.3. Antibacterial Activity of Marine Sponges

**Figure 2:**
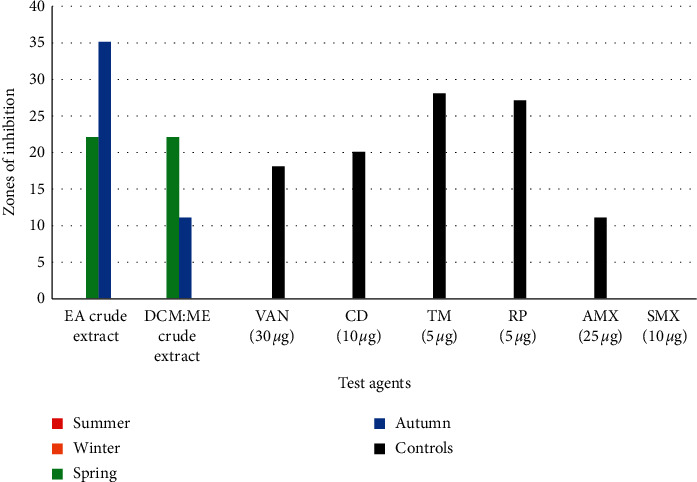
Antibacterial activity of ethyl acetate (EA) and dichloromethane : methanol (DCM : ME) crude extracts against MRSA.

*(1). Antimicrobial Susceptibility-Based Evaluations against Methicillin-Resistant S. aureus*. No bioactivity was observed during winter and summer. The EA crude extract (35 ± 04 mm) collected during autumn showed the best bioactivity compared to reference control antibiotics (vancomycin 30 *µ*g, 18 ± 1.2 mm; clindamycin 10 *µ*g, 23 ± 0.5 mm; trimethoprim 5 *µ*g, 28 ± 1 mm; rifampicin 5 *µ*g, 27 ± 0.1 mm) as seen in Figure 2.

**Figure 3:**
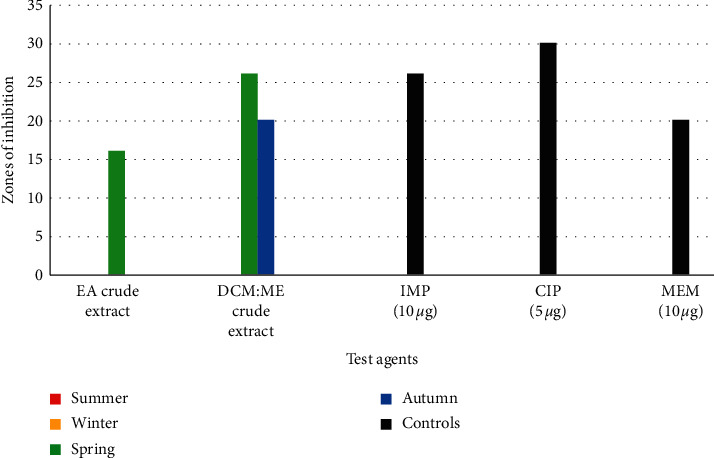
Antimicrobial activity of ethyl acetate (EA) and dichloromethane : methanol (DCM : ME) crude extracts against *P. aeruginosa*.

*(2). Antimicrobial Susceptibility-Based Evaluations against P. aeruginosa*. For activity against *P. aeruginosa* (Figure 3), no bioactivity was obtained during winter and summer. The highest inhibition zones were recorded with DCM : ME spring crude extracts of *Psammaplysilla* sp. 1 (26 mm) as seen in Figure 3, followed by crude extracts obtained from the autumn collection (20 ± 03 mm), while ethyl acetate crude extracts recorded lower inhibition zones (16 ± 0.2 mm). Significant differences in the mean zones of inhibition (*p*=0.0127) were observed against *P. aeruginosa* for collections from all four seasons. The antibiotic ciprofloxacin (30 ± 0.3 mm) performed slightly better than DCM : ME crude extract collected in spring (26 mm).

**Figure 4:**
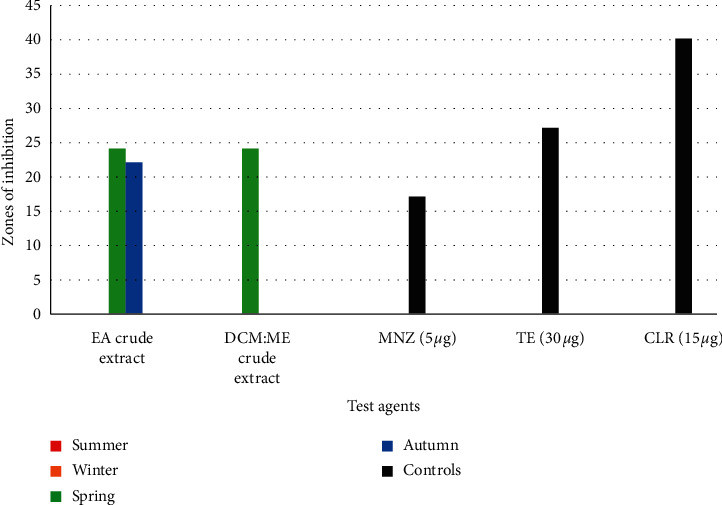
Antibacterial activity of ethyl acetate (EA) and dichloromethane : methanol (DCM : ME) crude extracts against *C. difficile*.

*(3). Antimicrobial Susceptibility-Based Evaluations against C. difficile*. Slightly similar bioactivities were observed seasonally with *Psammaplysilla* sp. 1 (22 mm and 24 mm) during autumn and spring, respectively, against *C. difficile* (Figure 4). A *p* value of 0.1342 was obtained for the mean zones of inhibition of the samples collected during all four seasons against *C. difficile*, thus illustrating no statistically significant differences in the bioactivity profiles of specimens collected during autumn and spring. This could also further imply that the efficacies of both EA and DCM : ME crude extracts collected in spring and autumn are similar.

**Figure 5:**
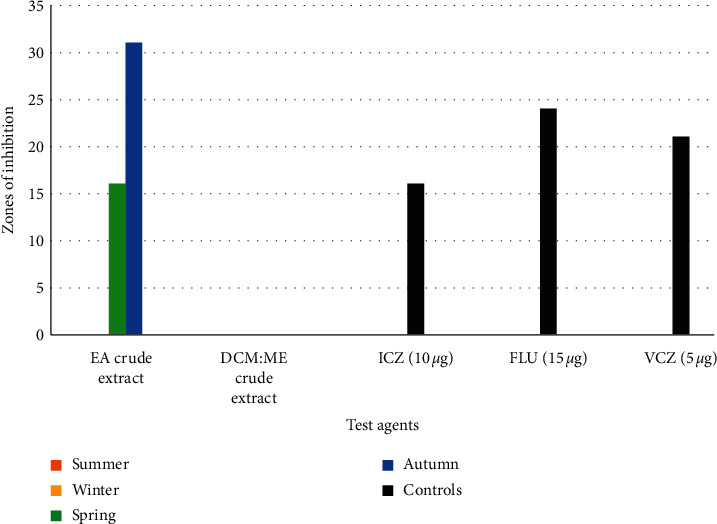
Antifungal activity of ethyl acetate (EA) and dichloromethane : methanol (DCM : ME) crude extracts against *A. fumigatus*.

*(4). Antimicrobial Susceptibility-Based Evaluations against A. fumigatus*. Only *Psammaplysilla* sp. 1 EA crude extracts collected during autumn and spring showed marked activity against *A. fumigatus*. Inhibition zones were 31 ± 0.1 mm and 16 mm in autumn and spring, respectively (Figure 5). Crude extracts of DCM : ME collected in all four seasons showed no activity against *A. fumigatus*. EA crude extracts obtained in autumn showed more significant bioactivity than all the standard antifungal drugs ICZ (16 ± 0.8 mm), FLU, 15 *µ*g (24 ± 2.1 mm), and VCZ, 5 *µ*g (21 ± 0.2 mm), as seen in Table 1. Statistically significant differences were observed in EA and DCM's bioactivity potentials DCM : ME crude extracts collected in spring and autumn against *A. fumigatus* (*p*=0.0164). This proves that seasons may play a role in sponges producing bioactive metabolites.

**Figure 6:**
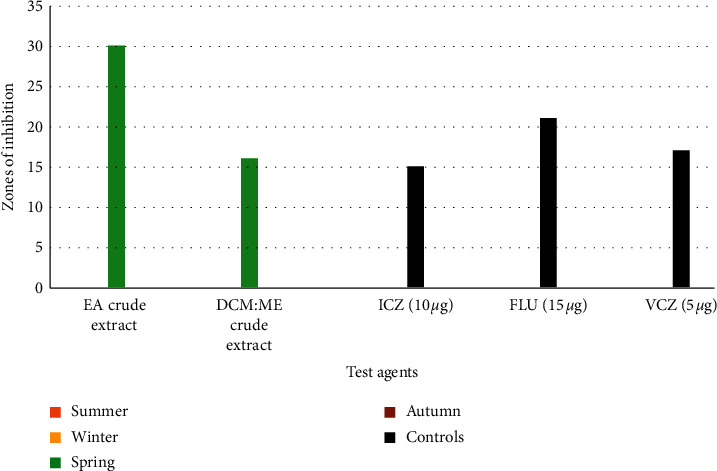
Antifungal activity of ethyl acetate (EA) and dichloromethane : methanol (DCM : ME) crude extracts against *C. albicans*.

*(5). Antimicrobial Susceptibility-Based Evaluations against C. albicans*. Only crude extracts collected in spring showed bioactivity against *C. albicans,* as seen in Figure 6. EA and DCM : ME crude extracts of *Psammaplysilla* sp. recorded zones of inhibition of 30 mm and 16 mm, respectively. *Psammaplysilla* sp. EA crude extracts bioactivity was higher than those recorded for the standard antifungal agents: ICZ (15 ± 0.1), FLU, 15 *µ*g (21 ± 0.2), and VCZ, 5 *µ*g (17 ± 0.3). Statistically significant differences (*p*=0.041) were observed in the mean zones of inhibition of crude extracts tested against *C. albicans*.

#### 3.1.4. Phytochemical Screening of Crude Extracts

*Psammaplysilla* sp. 1 that was tested for antimicrobial properties was also tested for the presence of seven (7) commonly reported phytochemicals. These phytochemical classes were also selected due to their antimicrobial properties. DCM : ME crude extracts tested positive for the presence of 2/7 of the phytochemicals (terpenoids and flavonoids), while EA crude extracts tested positive for 1/7 of the phytochemicals (saponins) as seen in Table 2.

#### 3.1.5. Gas Chromatographic Analysis of *Psammaplysilla* sp. 1

Ten antimicrobial compounds previously reported were present in the ethyl acetate crude extracts of *Psammaplysilla* sp. 1 as seen in Table 3. 1-Oxaspiro[2.5]octane-2-carbonitrile, methyl 10,11-tetradecadienoate, methyl 11,12-octadecadienoate, heptane, 3,3,5-trimethyl-phenol, 2,6-dibromo, and 2-heptacosane are compounds with antibacterial properties while heptane, 3,3,5-trimethyl and bicyclo[4.2.0]octa-1,3,5-trien-7-ol are known to exhibit antimicrobial properties; these compounds have been found to have antioxidant, anti-inflammatory, and antiviral properties. The antibacterial compound 2-heptacosane had the highest molecular weight. The compounds isolated further had different chemical structures except for methyl 10,11-tetradecadienoate and methyl 11,12-octadecadienoate.

### 3.2. Discussion

The vast nature of the marine environment favours a great deal of ecological diversity. Such ecosystems are home to a great diversity of important marine species such as sponges that have been reported to produce structurally diverse metabolites. Biological diversity is due to many physical and chemical parameters such as pH, temperature, salinity, and turbidity (Hamed et al., 2015). Differences in these parameters exert a driving force on the adaptive survival strategies, leading to the synthesis of new metabolites [1]. From 2014, approximately 30% of all marine natural products were isolated from marine sponges [34]. Because of this, significant interest in the discovery of new antimicrobials is tied to marine sponge research. Based on the results obtained from this study, turbidity recorded during spring could have a role in the antimicrobial activity observed against all test pathogens, as seen in Figures 2, 3, and 5.

According to the South African Water Quality Guidelines and WHO, a turbidity reading > one NTU indicates contaminated seawater [35]. Therefore, Zasloff [36] reported that the contamination could cause increased microbial pathogen interaction with the sponge species and yield high and probably a synthesis of diverse secondary metabolites notably during spring, as high bioactivities were recorded. The same could not be accounted for in winter as no bioactivity was observed during that season. However, such observation requires further validation as no studies have looked at the correlation between environmental parameters such as pH, temperature, turbidity, and salinity and the bioactivity potential of marine sponge.

Our crude extracts were tested for the presence of seven (7) commonly reported phytochemicals. Terpenoids and flavonoids were found in DCM : ME crude extracts, while EA crude extracts only tested positive for the presence of saponins. A study reported by Govinden-Soulange et al. [37]showed that two Mauritian sponge species *Stylissa* spp and *Biemnatubulosa* EA crude extracts contained saponins. Both sponges showed bioactivity against *S. aureus*, of which our collected sponge samples also showed bioactivity against MRSA. Another study was done by Fouad et al. [38], and it was observed that saponins obtained from marine sponge crude extracts only showed bioactivity against *C. albicans* and not MRSA.

Calabro et al. [39] further reported the same findings as their marine sponge *Poecillastra compressa* from the Mediterranean Deep-Sea contained saponins that only showed antifungal properties when tested against *A. fumigatus*. In our study, both antibacterial and antifungal activities were observed in the ethyl acetate crude extract, which contained saponins. Therefore, our findings contrast with those observed by the previous two studies. Our findings were further in agreement with the findings reported by Warad et al. [40] where the marine sponge *Callyspongia diffusa* crude extracts contained flavonoids that showed bioactivity against *C. albicans*.

Our *Psammaplysilla* sp. 1 crude extracts showed broad-spectrum bioactivity. These crude extracts were bioactive against both bacterial and fungal species, thus illustrating the efficacy or potential of the metabolites present within the sponge compared to the previously reported studies. Kim et al. [30] reported crude extracts found *Psammaplysilla* sp. 1 possessed bactericidal activity in *Staphylococcus aureus* strains (MRSA). Their study further reported the inactivity of some of the extracts against different Gram-negative bacteria strains such as *Escherichia coli*, *Pseudomonas aeruginosa*, *Salmonella typhimurium*, *Klebsiella oxytoca*, *Enterobacter cloacae*, and *Citrobacter freundii*. Our study observed different results as bioactivity was observed *Pseudomonas aeruginosa* (DCM : ME-26 mm). This could be due to our sample containing different types of bioactive metabolites within the same phytochemical class.

The bioactivity assays results obtained (Figures 2–6) from this study revealed that marine sponge species are capable of producing highly bioactive compounds effective against pathogenic microbes as previously reported by Putra et al. [41]. Sponges have been noted to produce a broad array of marine natural products [42] and possess significant chemical diversity [6, 43]. This can be further supported by the results of the crude extracts of *Psammaplysilla* sp (35 ± 0.4 mm), which performed better than control antimicrobials tested against three bacterial species (MRSA, *P. aeruginosa,* and *C. difficile*) and two fungal species (*A. fumigatus* and *C. albicans*). Our study observed that during winter and summer seasons, no bioactivity was observed against the test microorganisms except for spring and autumn seasons. This study is the first to report complete seasonal evaluations of the antimicrobial potentials *Psammaplysilla* species. No existing data were available on the seasonal bioactive profiles of *Psammaplysilla*. However, for other sponge's experimental data was available.

A study reported by Page et al. [44] revealed that wild *M. hentscheli* (sponges) exhibited a variation in the concentrations and efficacy of bioactive compounds (mycalamide, pateamine, and peloruside A) from different geographic regions and at different times of the year, thus illustrating that seasons do play a role in the different bioactivity profiles of marine sponges across various geographic locations. Devi et al. [45] from sponges *Clathria* sp. and *Axinella* sp. collected in South and East India during summer revealed no bioactivity for crude extracts of both species tested against *P. aeruginosa* and MRSA. The same observation was made with this study's crude extracts collected in summer as no bioactivity was recorded against MRSA and *P. aeruginosa*. Another study done by Cita et al. [3] on East Java Indonesian sponges collected during the rainy season revealed a different outcome as the same species (*Axinella* sp. and *Clathria* sp.) showed bioactivity against MRSA and *P. aeruginosa* as zones of inhibition of 15 ± 0.2 and 7 ± 0.21 were obtained, respectively, thus illustrating that different seasons of different biogeographic locations result in different bioactivity potentials being reported. In addition to this, our collected species were only bioactive during spring and autumn against *P. aeruginosa*, which are opposite seasons to those reported by Cita et al. [3] and Devi et al. [45].

Upon GC mass spectrometric analysis, several bioactive compounds previously reported were found in the crude extracts. Khatua et al. [28] isolated 2-Heptacosanea and methyl 11,12-octadecadienoatefrom *Trichosanthes dioica* root extract showed antibacterial activity against *Proteus mirabilis* and *Bacillus subtilis*. A study reported by Pangal et al. [33] revealed that tert-butyl-p-benzoquinone has antibacterial properties against *E. coli* and *S. aureus*. Even though these two studies reported antibacterial activity against different bacterial species than those used in our study, this further confirms the broad-spectrum antibacterial properties exhibited by *Psammaplysilla* sp 1. The compounds found in the sponge crude extract were like those reported to be found in plant extracts. Kumar and Pal [46] made a similar observation when they found that D-phytosphingosine, a corresponding 4-hydroxy analog of D-sphinganine, is a significant sphinganoid base found in both higher plants and many invertebrates.

Our study indicates that seasons may play a role in the variation of bioactivity potential of sponge species; this was also observed even for the same sponge species because of different geographical locations. Kanagasabhapathy et al. [47] reported that *Psammaplysilla* sp. collected from the Gulf Mannar in India showed bioactivity against *P. aeruginosa* (11 mm). This bioactivity was much less than the bioactivity results obtained in or study. The highest bioactivity recorded was 26 mm from a DCM : ME crude extract collected during spring. Our crude extracts efficacy was slightly less than trimethoprim (5 *µ*g) 28 ± 1 mm. These findings agree with the study done by McClintock and Gauthier [10], which reported that most orders of Demosponge show significant bioactivity even though bioactivity varied even amongst the same species located in different regions. Green [48] further explained that the variations reported in different studies were due to the different methodologies used for extraction and geographical locations. Our study further agrees with Thompson et al. [49], which revealed that the season has a role in sponge species' bioactivity potentials.

Therefore, it is inevitable that the season might have a direct role in the bioactivity potentials of sponge species. The biogeographical location further influences the chemical diversity and presence of bioactive compounds. Secondly, both ethyl acetate and dichloromethane : methanol (1 : 1) have comparable bioactive metabolite extraction potentials from *Psammaplysilla* sp. 1 tissue and therefore are both recommended extraction solvents for future studies. Nevertheless, more studies are needed further to validate these findings concerning the *Psammaplysilla* sp. 1 as this study is the first to report seasonal bioactivity this species. Our study also validated South African marine sponges' antimicrobial potential against infectious pathogens as previously reported by Zoraghi et al. [50] and Veale et al. [51]. Davies-Coleman et al. [52] reported that South African has an enormous biodiversity of marine sponges. Also, many of them produce a broad diversity of biologically active compounds that can also be used for different pharmaceutical applications, such as anticancer drugs [52, 53]. This study in the future will investigate the anticancer and antiviral properties of *Psammaplysilla* sp. 1-based crude extracts and the possible application in drug delivery systems.

## 4. Conclusion

Our study revealed that selected marine invertebrates such as sponges synthesize biologically active compounds against bacterial and fungal pathogens. The study has also identified Phillips Reef in Algoa Bay, Port Elizabeth, as a perfect environment worth exploring to discover marine natural products for antimicrobial application, spring and autumn being the best seasons for collecting marine species bioactive compound isolation. The scientific approach of this study was different from previous studies done in South Africa, as most look at the antimicrobial potential of microbial flora found on the sponges whilst those that look at the sponge matrix seek to isolate bioactive compounds for anticancer, antioxidant, and anti-inflammatory properties; from this work, a positive correlation can be made between the bioactivity potentials of certain sponge species with the season. A new sponge species was successfully identified as *Psammaplysilla* sp. 1. *Psammaplysilla* sp. 1 is an excellent source of potential antibacterial agents for treating bacterial and fungal-related infections as this species showed broad-spectrum bioactivity. This study further recommends evaluating the bioactive crude extracts for the constituents present in the crude extracts of *Psammaplysilla* sp. 1 for other biomedical applications than antimicrobial properties.

## Figures and Tables

**Table 1:** Physicochemical parameters of Phillips Reef in Algoa Bay.

Physicochemical parameters						
Season	Mean/CV	pH	O_2_ (mg L^−1^)	Temp (^o^C)	Sal (psu)	EC (mS cm^−1^)	TB (NTU)
Winter	Mean	8.18	7.72	16	35.47	44.4	1.62
	CV	0	0	0	0	0	0.0152

Spring	Mean	8.01	5.52	15.45	35.12	43.5	3.04
	CV	0	0.0018	0.0003	0	0	0.0502

Summer	Mean	8.32	5.97	17.86	35.12	45.9	2.05
	CV	0.05		0.0005	0.0004	0.0002	0

Autumn	Mean	8.78	6.85	20	35.22	48.2	1.05
	CV	0.0006	0.0029	0	0	0	0

M = mean; CV = coefficient of variability; O_2_ = oxygen; Sal = salinity; EC = electroconductivity; Temp = temperature; TB = turbidity.

**Table 2:** Phytochemical screen *Psammaplysilla* sp. 1 isolated from Phillips Reef, Port Elizabeth.

	Dichloromethane : methanol (1 : 1)							Ethyl acetate						
	1	2	3	4	5	6	7	1	2	3	4	5	6	7
*Psammaplysilla sp*. 1	−	−	+	−	−	+	−	−	−	−	−	+	−	−

1: tannins; 2: phenolics; 3: terpenoids; 4: anthraquinones; 5: saponins, 6: flavonoids; 7: alkaloids; +: present; −: absent.

**Table 3:** Gas chromatographic analysis of *Psammaplysilla* sp. 1 ethyl acetate crude extracts.

No	Organic compounds	Molecular weight	Chemical structure	Biomedical applications	References
1	Tetradecyl trifluoroacetate	144	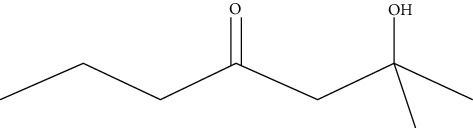	Antibiofilm activity	Gadhi et al. [25]

2	1-Oxaspiro[2.5]octane-2-carbonitrile	137	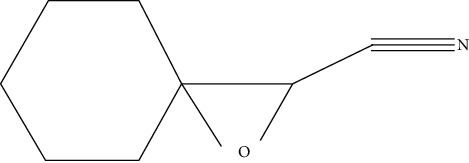	Antibacterial activity	Ibraheam et al. [26]

3	Methyl 10,11-tetradecadienoate	238	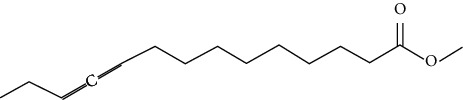	Antibacterial activity	Sharma et al. [27]

4	Methyl 11,12-octadecadienoate	294	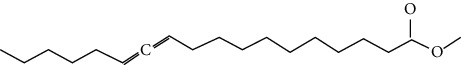	Antioxidant and antibacterial activities	Khatua et al. [28]

5	Heptane, 3,3,5-trimethyl-	142	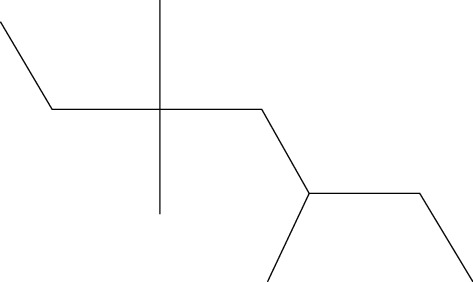	Anti-inflammatory, antifungal, and antibacterial activities	Kurashov et al. [29]

6	Bicyclo[4.2.0]octa-1,3,5-trien-7-ol	120	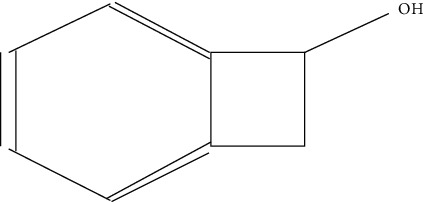	Antioxidant and antifungal	Kim [30]

7	Phenol, 2,6-dibromo-	250	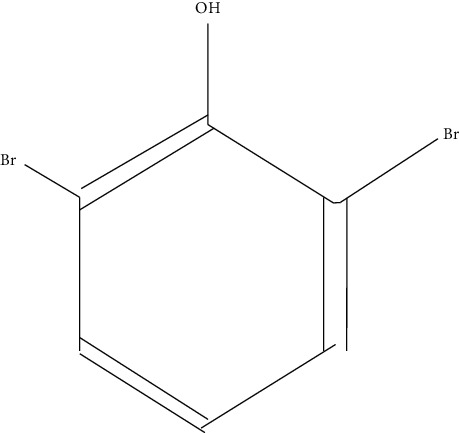	Antimicrobial, antioxidant, enzyme inhibitor	Sun et al. [31]

8	Benzyl alcohol, 4-fluoro-3-methoxy-	156	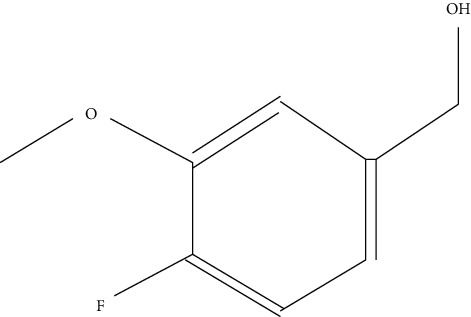	Antimicrobial activity	Wintola and Afolayan [32]

9	tert-Butyl-p-benzoquinone	220	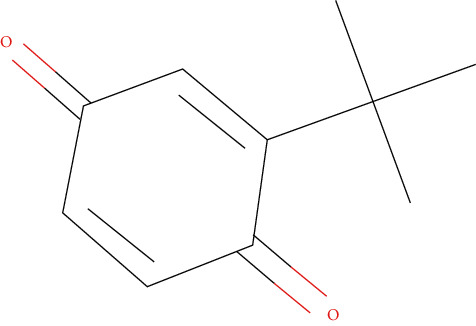	Antimicrobial, antiviral, anti-inflammatory	Pangal et al. [33]

10	2-Heptacosane	394	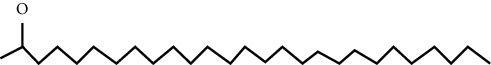	Antibacterial activity	Khatua et al. [28]

## Data Availability

All data used in this study are included within this article.
